# Fire ignition during laser surgery in pet rodents

**DOI:** 10.1186/1746-6148-8-177

**Published:** 2012-09-26

**Authors:** Tommaso Collarile, Nicola Di Girolamo, Giordano Nardini, Ivano Antonio Ciraci, Paolo Selleri

**Affiliations:** 1 Clinica per Animali Esotici, Centro Veterinario Specialistico, Via Sandro Giovannini 53, Rome, Italy; 2 Clinica Veterinaria Modena Sud, Spilamberto, MO, Italy

**Keywords:** Laser, Rodent, Pet, Surgery, Fire, Ignition, Face mask, Burn

## Abstract

**Background:**

Laser surgery is an attractive alternative to other means of section device in terms of tissue inflammation and interaction, which has been extensively used in human and veterinary medicine. Although accidental ignition during laser surgeries is sporadically reported in human medical literature, to the authors’ knowledge this is the first report regarding laser-dependent fire ignition during surgery in veterinary medicine.

**Case presentation:**

Two rodents, a 13-month old, 27-gram, male pet mouse (*Mus musculus*) and a 1-year old, female Russian hamster (*Phodopus sungorus*), underwent surgical removal of masses with diode laser. During the surgical procedures fires ignited from the face masks. The mouse presented severe burns on the head and both forelimbs, it was hospitalized and approximately 2 months after surgery burns were resolved. The hamster presented severe burns on the face and the proximal regions of the body. At 72 hours from the accident the hamster was euthanized.

**Conclusion:**

The present report suggests that fire ignition is a potential life-threatening complication of laser surgery in non-intubated rodents maintained under volatile anesthesia. High oxygen concentrations, the presence of combustible, and the narrowness of the surgical field with the face mask during laser surgery on rodents are risk factors for fire ignition.

## Background

Laser surgery is an attractive alternative to other means of section device in terms of tissue inflammation and interaction [1], which has been extensively used in human [2-5] and veterinary medicine [6-8].

In laboratory rodents, laser surgery promoted rapid postoperative healing [9] and allowed a significant reduction in tumor recurrence and mortality rates [10]. Several experienced surgeons propose the use of laser surgery in pet rodents [11,12].

Indeed, several articles on the complications associated with laser surgery have been published [13-15]. Among other complications (e.g. accidental burns, edema of mucus membranes and airway obstruction in larynx surgery), ignition is probably the most dangerous one [16,17].

Although some authors reported fire ignition as a risk associated with laser procedures [18], to the authors’ knowledge specific reports of laser-dependent fire ignition in veterinary medicine are lacking. The objective of this report is to describe two cases of fire ignition observed during laser surgery in non-intubated rodents under volatile anesthesia.

## Case presentation

### Case 1: Mouse

**Figure 1:**
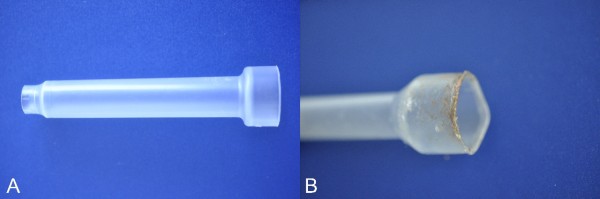
**Face mask employed by the authors for maintenance of volatile anesthesia in rodents.** The face mask in figure A, due to the absence of a diaphragm that permits to achieve a seal on the animal’s nose or neck, did not prevent leakage of oxygen and anesthetic gasses. In figure B it is depicted the face mask used for anesthesia of the mouse after fire ignition. The use of tight-fitting face masks providing a hermetical seal seems necessary during laser surgery under volatile anesthesia in non-intubated rodents.

A 13-month old, 27-gram, male pet mouse (*Mus musculus*) was presented for the evaluation of a growing mass located in the right ear pinna. A thorough physical examination was performed and no other abnormalities were found. Due to the need of anesthesia to perform any additional diagnostic evaluation, direct removal of the mass by use of surgical laser was proposed. Anesthesia was induced with 8% sevoflurane (Sevorane, Abbott, UK) administered via an induction chamber. Anesthetic maintenance was facilitated with 4.5-5% sevoflurane and 0.5 litre/minute oxygen delivered via a small, modified, face mask (Figure 1). The mouse was laid supine and retained spontaneous ventilation throughout surgery. Surgical scrub was performed with chlorhexidine (Clorexyderm, ICFpet, Palazzo Pignano, Italy) and sterile saline. A diode laser (Veilure S9, Lasering S.r.l., Modena, Italy) transduced by an optic fiber was used in contact [19], continuous mode at 3 W to excise the mass. During surgical excision of the mass an accidental fire ignited from the face mask. Emergency procedures for free fire were adopted: the primary surgeon moved immediately the mouse away from the face mask. The anesthetist turned off the oxygen and the volatile anesthesia. The assistant surgeon poured water on the mouse. These procedures were executed almost at the same time. Thus in few seconds the fire was extinguished.

**Figure 2:**
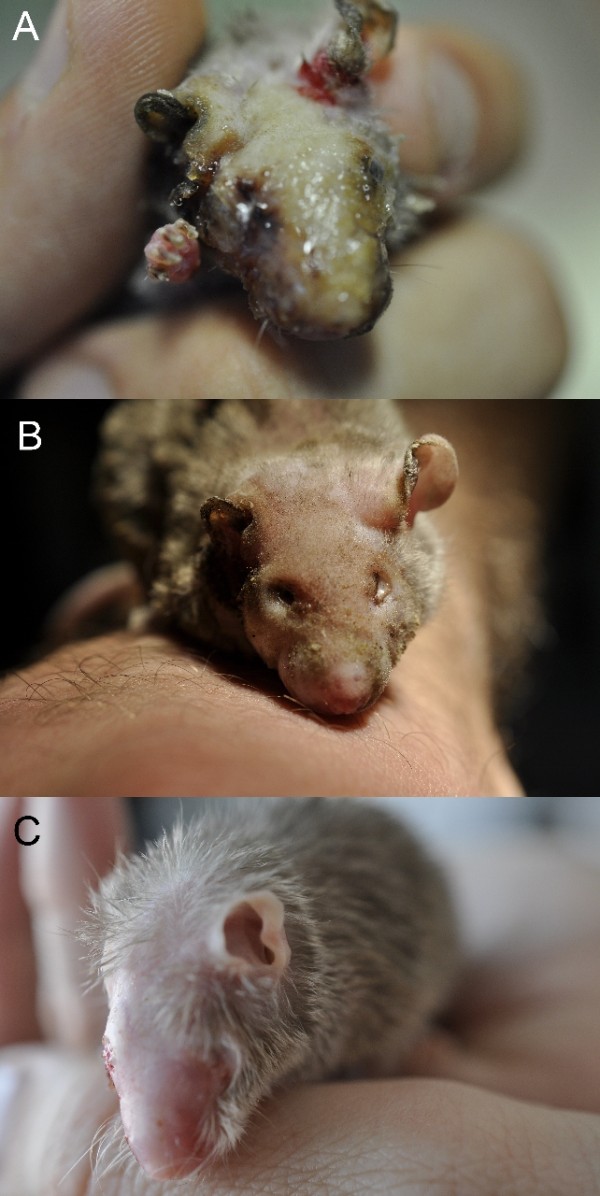
**Clinical progression of burn secondary to laser-dependent ignition of anesthetic gasses in a pet mouse.** The mouse at day 5 (A), 30 (B) and 60 (C) after fire ignition.

Nevertheless, the mouse presented severe burns on the head and both forelimbs (Figure 2A). The animal was hospitalized and standard medical measures for burns were administered. In the days following the surgery the mouse body weight dropped to 21 grams. Mouse’s clinical conditions slowly improved and four weeks following the accident it was discharged (Figure 2B). The mouse gradually returned to its initial weight and approximately 2 months after surgery the burns were resolved (Figure 2C).

### Case 2: Hamster

**Figure 3:**
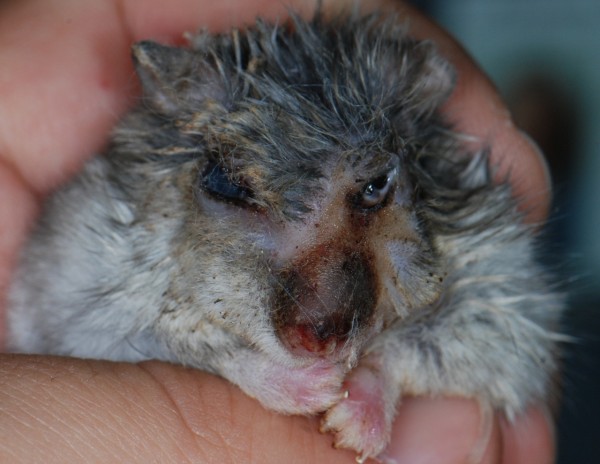
**Photograph of a pet hamster after that fire ignited during laser surgery.** Notice the spread of the lesion along the face.

A 1-year old, female Russian hamster (*Phodopus sungorus*) was presented for eyelid swelling and lameness. Physical examination revealed a firm, encapsulated mass located in the inner surface of the left upper eyelid and a round, pedunculated, 1-mm mass dorsally to the first finger of left forelimb. Surgical removal of both masses was elected. Anesthesia was induced with 5% isoflurane (Isoba, Schering-Plough, UK) administered via an induction chamber. After induction the hamster was maintained on 2.5-3% isoflurane with a modified face-mask. Trichotomy and surgical scrub with chlorhexidine (Clorexyderm, ICFpet, Palazzo Pignano, Italy) were performed around the two masses. The hamster’s eye was covered with moist gauze sponges in order to protect the corneas from damage by exposure to the laser beam. A 400 micron transducer of the diode laser (Veilure S9, Lasering S.r.l., Modena, Italy) was used to excise the palpebral mass. The transducer was settled on 1.5 W, non-continuous mode (On: 10 milliseconds, Off: 50 milliseconds). The mass on the eyelid was removed without adverse reactions during the procedure. Diode laser was then used in continuous mode, 3.5 W, to excise the mass on the left forelimb. After few seconds from the beginning of the procedure, some sparks were seen originating from the laser beam and fire ignite from the face mask. Although the fire was immediately extinguished, the hamster presented severe burns on the face and the proximal regions of the body. Left eye vision was impaired due to corneal burn (Figure 3). The hamster was hospitalized and aggressive supportive therapy was initiated. Nevertheless there was a rapid worsening of its clinical status. At 72 hours from the accident the hamster was humanely euthanized.

## Discussion

The present report underline that ignition is a potential life-threatening complication of laser surgery in non-intubated rodents under volatile anesthesia. Although accidental ignition during laser surgeries is sporadically reported in human medical literature [17,20-23], to the author’s best knowledge this is the first report in which laser-secondary ignition is described in veterinary literature.

For any fire to start, three elements must be present: heat or an ignition source, fuel and an oxidizer. The three elements constitute the so-called “fire triad” or “fire triangle” [24,25]. An oxidizer is a substance that gains electrons in a red-ox chemical reaction. The most common oxidizers in the operating room are oxygen and nitrous oxide [25]. The most common ignition sources cited in operating room fires seem to be the electrosurgical unit [24], although also lasers are another common ignition source. As previously stated heat can replace the ignition source: the heat produced by the fiberoptic light source was responsible of operating room fire [26]. Fuels can be from alcohol based solutions, to any cloth-containing or paper-containing materials on or around the patient, including abdominal gasses [24].

Indeed, an oxygen-rich atmosphere, flammable materials, and ignition sources are virtually ubiquitous in the modern-day operating room [24].

In human medical literature, laser-secondary ignitions are mainly reported during upper airways surgeries [20], especially laryngeal [17,22,23] and tracheal [21] surgeries. On 20,000 laryngeal laser-surgical procedures, Sesterhenn and colleagues [27] reported 15 cases (0.075%) of tube fires. The mechanism of combustion is the initial penetration of the endotracheal tube by the laser and then the ignition of the tube. This is facilitated by the heat produced by the laser, the flow of oxygen, inflammable anesthetic agents or combustible materials [16,23,28]. We suppose that in the present cases the elements composing the “fire triangle” (oxidizer/inflammable substance/ignition source) [25] were: 100 per cent oxygen, sevoflurane or isoflurane and diode laser at 3–3.5 W continuous pulsation, respectively. Decrease or removal of any element of the fire triangle could have prevented fire ignition.

In a recent study 15 rats underwent resection of a 3-mm transverse area of the anterior tongue by use of a carbon dioxide laser [29]. The rats in the study were not exposed to inhalant anesthesia or to oxygen. It is not unexpected that no fire ignition consequent to the procedure was reported in that study. This finding corroborates the role that oxygen and inhalant gasses play in fire ignition during laser surgery.

In the present work we presented two cases in which the oxygen was administered at 100 per cent concentration. Oxygen concentrations of 50 and 75 per cent did not alter the time to ignition of surgical patties exposed to laser beam. Indeed the time to ignition felt significantly when the oxygen concentration was further increased to 100 per cent [30]. A previous study already demonstrated that surgical drapes present a lower time to ignition when exposed to higher oxygen concentration [31]. Considering such findings, ventilation with 100 per cent oxygen should be avoided when laser surgeries are performed under face mask ventilation. An alternative to 100 per cent oxygen ventilation may be the ventilation with a lower oxygen concentration (e.g. 30-60-80 per cent), as it has been suggested in human medicine to prevent atelectasis [32-34]. Although lower oxygen concentrations may decrease the risk of fire ignition, specific reports investigating its impact on rodent surgery outcome are necessary before its application in clinical practice.

Intubation in rodents is, in most cases, not easily performed; therefore anesthesia is often maintained with a tight-fitting face mask connected to the breathing system [35,36]. As suggested by Dave and Mahaffey [37] “*The use of face masks and nasal cannulae should be avoided as there is always some leakage around these devices*”. In the present reports the face mask employed (Figure 1) did not prevent leakage of oxygen and anesthetic gasses. Face masks similar to those employed by the authors are often used during surgeries of small exotic animals [35,38,39]. The use of tight-fitting face masks providing a hermetical seal seems necessary during laser surgery under volatile anesthesia in rodents. Anesthetic gas leakage from standard rodent non-rebreathing circuits has been demonstrated [40]. The use of modified face masks significantly reduced the volume of gas leakage. The masks were modified by addition of a latex diaphragm to the conical mask attached to the Mapleson E circuit [40]. Nevertheless, before laser can be safely employed on non-intubated animals under volatile anesthesia, specific trials focusing on the real efficacy of face masks in preventing gas leakage are necessary.

In the two cases described topical scrub was not performed with alcohol due to its intrinsic inflammable properties. Unfortunately, also most commercial chlorhexidine solutions contain alcohol, and their flammability is in direct proportion to their alcohol concentration [24]. Although some case reports identifying alcohol-based skin preparations as a fuel source in surgical fires have been published in human medical literature [41-43], a recent study was unable to demonstrate flammability of isopropyl alcohol exposed to electrofulguration in experimental settings [44]. Conversely, when materials in the study were poured with chlorhexidine gluconate under saturated and damp conditions produced a spark and associated mild to moderate char [44]. Only water-based prep solutions such as betadine contain no alcohol and, therefore, can be considered truly nonflammable [24]. It should be also mentioned that the use of alcohol in surgeries of rodents is discouraged, as it could promote hypothermia [11,45].

## Conclusions

Although in human medical literature most cases of fire ignition seems to be managed without vital damage of the patient [22,46], in the present cases, catastrophic, life-threatening injuries were suffered from both animal patients.

Fire ignition is a potential complication during laser surgeries in rodents due to the narrowness of surgical field with oxygen outflow and anesthetic gasses. Besides paying appropriate care when specific regions (e.g. airways and oral cavity [7,8]) are target of laser surgery, the surgeon should always be aware of fire ignition when laser surgeries are performed on non-intubated animals under volatile anesthesia.

### Consent

Consent was obtained from the owners of the animals for publication of this case report and any accompanying images.

## Competing interests

The authors declare that they have no competing interests

## Authors’ contributions

TC and GN were the surgeons of the mouse and the hamster, respectively. ND was the assistant surgeon of the first case and drafted the manuscript. IC was the anesthetist of the first case and took care of the mouse during its recovery. PS conceived the report and helped to draft the manuscript. All authors read and approved the final manuscript.
